# hnRNPH1 Inhibits Influenza Virus Replication by Binding Viral RNA

**DOI:** 10.3390/microorganisms13010024

**Published:** 2024-12-26

**Authors:** Ruixue Xue, Danqi Bao, Tianxin Ma, Shiqi Niu, Zihua Wu, Xuehua Lv, Yunxiang Zhang, Guanlong Xu, Dawei Yan, Zhifei Zhang, Xue Pan, Minghao Yan, Qiaoyang Teng, Chunxiu Yuan, Zejun Li, Qinfang Liu

**Affiliations:** 1 Shanghai Veterinary Research Institute, 518 Ziyue Road, Minhang District, Shanghai 200241, China; ruixue0114@163.com (R.X.); baodanqi@westlake.edu.cn (D.B.); matianxin12@163.com (T.M.); 15837283120@163.com (S.N.); wuzihua6@163.com (Z.W.); ylanfanghua@163.com (X.L.); 13507656505@163.com (Y.Z.); yandawei@shvri.ac.cn (D.Y.); nzhangzhifei@163.com (Z.Z.); panxue@shvri.ac.cn (X.P.); m17519478956@163.com (M.Y.); tengqy@shvri.ac.cn (Q.T.); yuanchx@shvri.ac.cn (C.Y.); 2 Shandong Provincial Center for Animal Disease Control and Prevention (Shandong Provincial Center for Zoonoses Epidemiology Investigation and Surveillance), 4566 Tangye West Road, Licheng District, Jinan 250100, China; 3 China Institute of Veterinary Drug Control, Beijing 100082, China; xuguanlongw@163.com

**Keywords:** hnRNPH1, influenza virus, RNA recognition motif

## Abstract

During the life cycle of the influenza virus, viral RNPs (vRNPs) are transported to the nucleus for replication. Given that a large number of progeny viral RNA occupies the nucleus, whether there is any host protein located in the nucleus that recognizes the viral RNA and inhibits the viral replication remains largely unknown. In this study, to explore the role of hnRNPH1 in influenza virus infection, we knocked down and over-expressed the hnRNPH1 proteins in 293T cells, then infected the cells with the influenza virus. The results showed that the host hnRNPH1 inhibits the replication of H1N1 and H9N2 influenza viruses by restraining the polymerase activity of viruses. hnRNPH1 contains two RNA recognition motifs (RRM1) and RRM2. Further studies indicated that hnRNPH1 specifically binds to the viral RNA of the PB1, PA, and NP genes. Mutation of the key residues tryptophan and tyrosine in RRM1 and RRM2 abolished the binding affinity to viral RNA and the suppression of polymerase activity of the influenza virus. All the results suggested that hnRNPH1 suppresses polymerase activity and replication of the influenza virus by binding viral RNA.

## 1. Introduction

The influenza virus belongs to the genus influenza virus of positive Orthomyxoviridae, and its genome is single-stranded, negative-sense RNA. According to the antigenicity of nuclear protein (NP) and matrix protein (M1), influenza viruses are divided into four types: A, B, C, and D [1]. Type D influenza virus has been identified in cattle and pigs recently [2]. Influenza A virus poses the most serious threat to humans and animals. Several influenza pandemics throughout history were caused by the influenza A virus. The influenza A virus contains 18 HA and 11 NA subtypes based on the antigenicity of hemagglutinin (HA) and neuraminidase (NA). The H17N10 and H18N11 subtypes have been identified in bats [3].

Most RNA viruses replicate in the cytoplasm of cells, whilst the replication and transcription process of influenza virus occurs in the nucleus of the host cell [1]. During the life cycle of the influenza virus, vRNPs are transported from the cytoplasm to the nucleus [4]. The PB2 protein has the function of catching the 5′ end cap of host mRNA, and the PA protein cuts the RNA after the cap structure and connects it to the viral RNA [5]. The PB1 protein uses this as a primer to catalyze the synthesis of the mRNA chain and extend to the 5′ end of the viral (-) RNA. The newly synthesized mRNA exports the nucleus for the translation process. On the other hand, it replicates the (-) RNA genome into fully complementary RNA (cRNA) and then uses this as a template to replicate a large number of progeny viral RNA [6]. The newly synthesized PB1, PB2, PA, and NP proteins tightly wrap the viral genome to form RNPs, which are exported from the nucleus to the budding site on the cell membrane with the assistance of the M1 and NS2 proteins [7].

Given that a large amount of foreign RNA occupies the nucleus during the replication of the influenza virus, whether there is any host protein located in the nucleus that recognizes viral RNA and inhibits the viral replication remains largely unknown. The nuclear heterogeneous ribonucleoprotein (hnRNP) family is a multi-functional RNA-binding protein containing more than 20 proteins [8]. The main function of these proteins is to regulate the metabolism of mRNA precursors, including regulating the 3′ end modification of mRNA to enhance or reduce the stability of mRNA, regulating mRNA splicing. Studies have shown that certain hnRNPs are important for virus replication in cells [9,10]. Matteo Scalabrin et al. reported that hnRNP A2/B1 enhances HIV-1 transcription by unfolding LTR promoter G-quadruplexes [11]. Studies have shown that hnRNP C1/C2 promotes DENV replication during the stage of viral RNA synthesis [12,13], and DENV-2 and JUNV induce hnRNP K cytoplasmic translocation to improve viral multiplication [13]. HnRNP K/NS1-BP mediates influenza virus M splicing to support viral replication [14]. In contrast, hnRNP K has the opposite function for other viruses. Liu et al. reported that hnRNP K negatively regulates FMDV Translation and Replication as a novel internal ribosomal entry site-transacting factor [15]. HnRNP A1 inhibits the replication of the Porcine Epidemic Diarrhea Virus by interacting with the viral Nucleocapsid Protein [16]. HnRNP L negatively regulates FMDV replication by inhibiting viral RNA synthesis [17].

hnRNPH1, as an RNA-binding protein, plays an important role in pre-mRNA processing, metabolism, and transport. Recently, Philipp Schult et al. reported that the hnRNPH1 binds to the genomic RNA of yellow fever virus (YFV), and the YFV infection recruits hnRNPH1 to the cytoplasm from the nucleus; the relocated hnRNPH1 helps reduce the cell stress caused by infection, subsequently supporting the virus replication [18]. Given that the influenza virus replicates in the nucleus of the host cell, the aim of this study is to explore whether hnRNPH1 influences the replication of the influenza virus.

In this study, we found that overexpressed hnRNPH1 inhibits the replication of the influenza virus, and interference with the expression of hnRNPH1 protein promotes the replication of the influenza virus, which suggests that the hnRNPH1 is a negative regulator of influenza virus replication. Further studies have shown that the hnRNPH1 protein inhibits the influenza virus polymerase activity of H1N1 and H9N2 viruses. hnRNPH1 contains two RNA recognition motifs (RRM1) and RRM2 [19]. The present data indicated that hnRNPH1 specifically binds to PB1, PA, and NP RNA. Mutation of the key residues tryptophan and tyrosine in RRM1 and RRM2 abolishes the binding affinity to viral RNA and the suppression of polymerase activity of influenza virus. All the results suggest that hnRNPH1 suppresses the polymerase activity and replication of the influenza virus by binding viral RNA.

## 2. Materials and Methods

### 2.1. Cells, Viruses, and Plasmids

The 293T, A549, and Hela cells were cultured in Dulbecco’s modified Eagle’s medium (DMEM), 10% heated-inactivated fetal bovine serum (Gibco, Grand Island, NE, USA), and 100 units per ml Pen/Strep antibiotics. MDCK cells were cultured in Minimum Eagle’s medium (MEM), 8% heated-inactivated fetal bovine serum (Gibco), and 100 units per ml Pen/Strep antibiotics. All cells were maintained at 37 °C with 5% CO_2_. Influenza A/WSN/33/H1N1virus (WSN/H1N1) was propagated and titrated in the MDCK cells.

The open reading frames (ORFs) of hnRNPH1 were amplified from the cDNA of A549 cells and cloned into the pCMV-N-FLAG vector, and the ORFs corresponding to the qRRM1, qRRM1 + qRRM2, and qRRM1 + qRRM2 + qRRM3 deleted regions of hnRNPH1 were constructed into the pCMV-N-FLAG vector. The plasmid expressing two amino acid changes (WY/AA) in the hnRNPH1 qRRM1 region was generated by PCR-based site-directed mutagenesis, using the pCMV-N-FLAG-hnRNPH1 vector as a template. In the same way, the plasmids expressing two amino acid changes (FY/AA) in the hnRNPH1 qRRM2 region and four amino acid changes (WYFY/AAAA) in the hnRNPH1 qRRM1 and qRRM2 regions were generated, respectively.

### 2.2. Immunofluorescence and Confocal Microscopy

The A549 cells and Hela cells grown on coverslips were infected with the influenza A virus WSN strain at a multiplicity of infection (MOI) 0.01. At the indicated time points, the cells were fixed with 4% paraformaldehyde. After treating the fixed cells with 0.3% Triton X-100, the cells were subjected to immunofluorescence staining, as previously described [20]. Briefly, the fixed cells were blocked with 5% bovine serum albumin and incubated with mouse anti-hnRNPH1 antibody (Sigma, New York, NY, USA) and rabbit anti-NP antibody (Abcam128193, Abcam, Shanghai, China). The fluorescence-conjugated antibodies (Alexa Fluor 488-conjugated anti-mouse and Alexa Fluor 555-conjugated anti-rabbit; Life Technologies, Carlsbad, CA, USA) were applied to detect primary antibodies. Nuclei were stained with 4′6-diamidino-2-phenylindole (DAPI). The location of the indicated proteins and nuclei was then observed using a ZEISS LSM510 META confocal microscope (Oberkochen, Germany).

### 2.3. Minigenome Assay

In order to measure the influenza virus polymerase activity, pCAGGS expression plasmids encoding PB1 (0.1 μg), PB2 (0.1 μg), PA (0.1 μg), and NP (0.1 μg) from each virus (H9N2, pH1N1, or WSN) were transfected into 200,000 293T cells using Lipofectamine 3000 (Thermo Fisher, Waltham, MA, USA) at ratios of 3 μL regent per μg plasmid DNA. As reporter constructs, we transfected 0.1 μg Poll-luc, which encodes a minigenome containing a firefly reporter flanked by influenza A promoter sequences. pCMV-Renilla luciferase (0.02 μg) was transfected as an internal reference. For exogenous expression, 0.1 μg of the relevant Flag-tagged hnRNPH1 gene, different hnRNPH1 mutant genes, or empty Flag, were co-expressed with the RNP components. At least 30 h post-transfection, the cells were lysed in 100 μL passive lysis buffer (Promega, Madison, WI, USA), and the dual-luciferase reporter assay kit (Promega) was used to measure bioluminescence on a FLUOstar Omega plate reader (BMG Labtech, Ortenberg, Germany). All minigenome assays were repeated in triplicate at least three times.

### 2.4. Expression and Purification of hnRNPH1 and Mutant RRM1+RRM2-WYFY/AAAA

The recombinant pET30a-hnRNPH1 or RRM1+RRM2-WYFY/AAAA plasmid was transformed into BL21 cells. A single colony was inoculated into 2.5 mL of LB with 100 μg/mL of kanamycin and incubated at 37 °C in a shaker at 225 rpm overnight. The next day, the overnight culture was added into 250 mL of 2-YT broth (Invitrogen, Waltham, MA, USA) and grown to OD 0.6 at 37 °C. Protein expression was induced with 1.0 mM isopropy-β-D-thiogalactoside (IPTG) at 20 °C for 6 h. Bacteria were pelleted and re-suspended in 25 mL of cold lysis buffer [20 mM HEPES, pH 7.5, 1 mM EDTA, 300 Mm NaCl, and 1× protease inhibitor cocktail (Roche, Basel, Switzerland)]. Bacteria were lysed at 8000 rpm through an EmulsiFlex-C5 homogenizer (Avestin, Ottawa, ON, Canada), and then the lysates were centrifuged at 13,000× *g* for 10 min. The supernatant was incubated with 2 mL His- beads at 4 °C for 1 h, and beads were washed 10 times with cold lysis buffer. His-hnRNPH1 or RRM1+RRM2-WYFY/AAAA was eluted with 250 mM imidazole (pH 8.0).

### 2.5. Electrophoretic Mobility Shift Assay (EMSA)

The vRNA of PB2, PB1, PA, and NP genes was transcribed in vitro using the T7 Quick High Yield RNA Transcription Kit (Beyotime, Shanghai, China) according to the manufacturer’s instructions. The hnRNPH1 and mutant proteins were expressed and purified in BL21 cells. To test the binding activity of hnRNPH1 to vRNA of PB2, PB1, PA, and NP genes, an electrophoretic mobility shift assay was performed using an EMSA kit (Beyotime, Shanghai, China), according to the manufacturer’s instructions. Briefly, the 1 μg vRNA samples were incubated with 0–4 μg purified hnRNPH1 or BSA proteins for 60 min at 23 °C in EMSA/Gel-Shift buffer containing RNase Inhibitor (0.2 units/μL) and protease inhibitor (1 units/μL). Then, the samples were stained with GOLDVIEW (Beyotime, Shanghai, China) and separated using 3% Agarose gel. Then, the results were detected using a Tanon 5200 Multi camera (Tanon, Shanghai, China) under a UV light.

### 2.6. Statistical Analysis

All data in this study were obtained from three independent repeats and analyzed using analysis of variance (ANOVA) in GraphPad Prism version 6.0 (GraphPad software Inc., San Diego, CA, USA). The differences are considered significant at *p* < 0.05 and extremely significant at *p* < 0.01.

## 3. Results

### 3.1. Identification of hnRNPH1 to Be a Negative Regulator for Influenza Virus Replication

**Figure 1:**
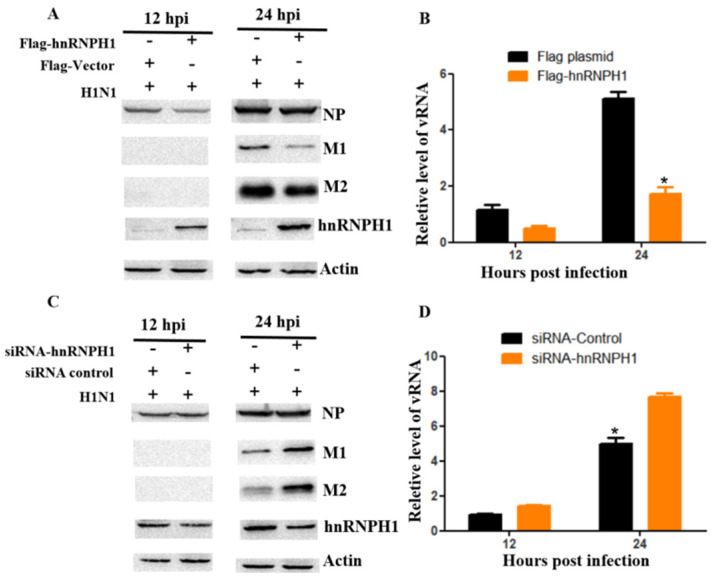
hnRNPH1 is a negative regulator for influenza virus replication. Overexpressed hnRNPH1 protein inhibited influenza virus replication at the levels of viral protein and vRNA (**A**,**B**); Knocking down hnRNPH1 protein improved influenza virus replication at the levels of viral protein and vRNA (**C**,**D**). * indicates significant difference between control and treated group (*p* < 0.05).

In this study, we observed that the overexpression of hnRNPH1 in cells inhibits the replication of the influenza virus. The levels of NP, M1, and M2 protein expression were decreased in hnRNPH1 overexpressed cells at various time points post-inoculation (Figure 1A). The viral load in the supernatant of hnRNPH1 overexpressed cells was lower than that of mock cells (Figure 1B). To further confirm whether hnRNPH1 affects influenza virus replication, hnRNPH1 was knocked down by siRNA on 293T cells, followed by inoculation of the H1N1 virus at an MOI of 0.01. Western blot analyses of lysates showed that the levels of viral NP, M1, and M2 proteins were higher in hnRNPH1 knockdown cells than in control cells (Figure 1C). The levels of viral load in the supernatant were higher in the hnRNPH1 knockdown cells at various time points post-inoculation compared with those in the control cells (Figure 1D). Taken together, our findings suggest that hnRNPH1 acts as a negative regulator for influenza virus replication.

### 3.2. hnRNPH1 Co-Localized with vRNPs in Nucleus of Infected Cells

**Figure 2:**
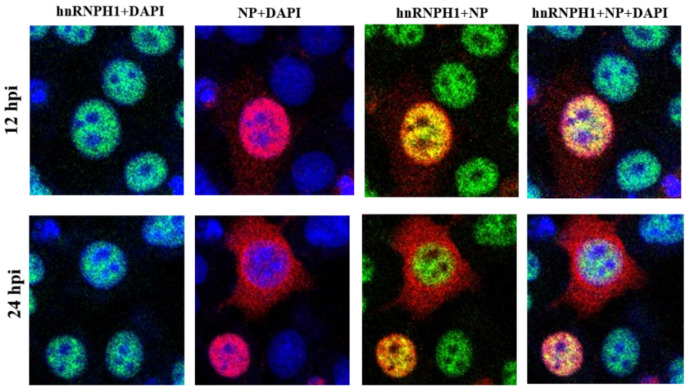
hnRNPH1 co-localized with NP protein in the nucleus of infected cells. Co-localization of NP and hnRNPH1 protein in H1N1 infected MDCK cells at early (12 hpi) and late (24 hpi) stages of infections.

Given that the vRNPs of influenza viruses are localized in the nucleus during the early stage of infection, the co-localization of hnRNPH1 and vRNPs was checked in infected cells. The results showed that hnRNPH1 co-localized with the NP protein in the nucleus during the early stage of influenza virus infection. While the NP protein associated with vRNPs was transported out of the nucleolus for progeny virus assembly, hnRNPH1 still retained in the nucleus, which suggested that hnRNPH1 co-localized with the vRNPs in the nucleolus during the early stage of the influenza virus replication (Figure 2).

### 3.3. hnRNPH1 Inhibits Viral RNA Polymerase Activity In Vitro

**Figure 3:**
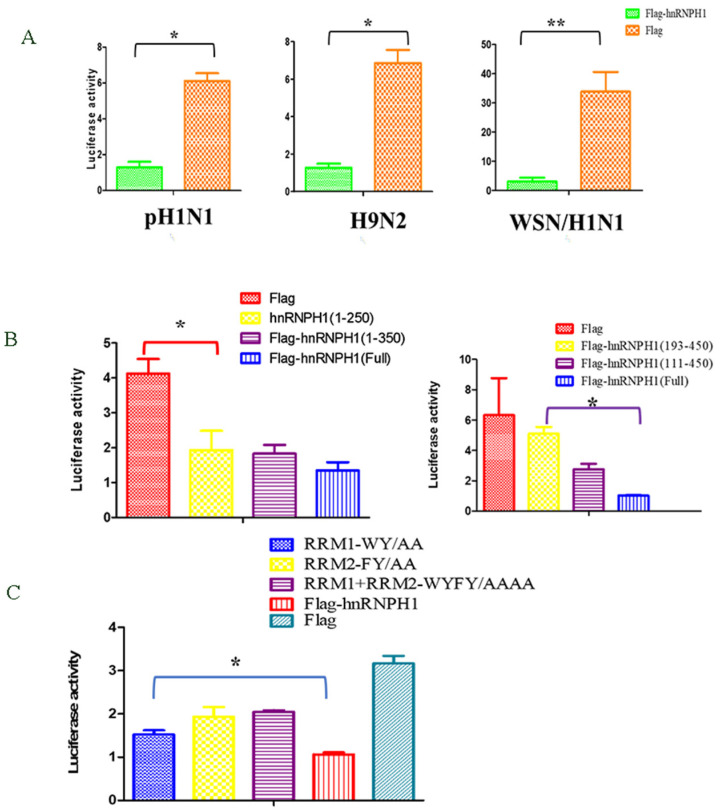
hnRNPH1 inhibits viral RNA polymerase activity of the influenza virus in vitro. hnRNPH1 inhibited the polymerase activity of pandemic H1N1 (pH1N1), WSN/H1N1, and H9N2 (**A**). Influence of different truncated hnRNHPH1 on polymerase activity of WSN/H1N1 (**B**). Influence of RRM1 and RRM2 mutated hnRNHPH1 on polymerase activity of WSN/H1N1 (**C**). * indicates significant difference between control and treated group (*p* < 0.05). ** *p* < 0.01.

Given that hnRNPH1 is a nuclear-localized protein and to explore whether hnRNPH1 influences the polymerase activity of vRNPs of influenza viruses, a minigenome assay was conducted, as described previously. The results showed that hnRNPH1 could significantly inhibit the viral RNA polymerase activity of H9N2, pH1N1, and WSN/H1N1 in vitro (Figure 3A), which confirmed that hnRNPH1 is a negative regulator for influenza virus replication. To further map the domain(s) of hnRNPH1 that is responsible for the inhibition of polymerase activity, different truncated hnRNPH1 expression plasmids were constructed and tested using the minigenome reporter assays. The results showed that hnRNPH1 (1–250 aa), (1–350 aa), and (111–450 aa) truncated protein still inhibited viral RNA polymerase activity, whilst hnRNPH1 (193–472 aa) failed to inhibit viral RNA polymerase, which indicated that the N-terminal domain 111–193 aa is mainly responsible for inhibiting influenza virus replication (Figure 3B).

The hnRNP H family proteins contain two RNA recognition motifs (RRM1) and RRM2 located in the N terminal. In RRM1 and RRM2, four functional residues, tryptophan (W) and tyrosine (Y) in qRRM1 and phenylalanine (F) and tyrosine (Y) in qRRM2 of hnRNPH1, determine the RNA-binding ability. Mutations of those residues significantly reduced the RNA-binding capacity of hnRNP proteins [19]. To further explore whether these four amino acids are crucial for the inhibition of hnRNPH1 on the polymerase activity of the influenza virus, three hnRNP mutants, RRM1-WY/AA (W and Y amino acids were both mutated into A), RRM2-FY/AA (F and Y amino acids were both mutated into A), and RRM1+RRM2-WYFY/AAAA expression plasmids were generated and subjected to minigenome reporter assays. The results showed that all three hnRNPH1 failed to inhibit viral RNA polymerase activity compared with the parental hnRNPH1 (Figure 3C), which suggested that the hnRNPH1 inhibited the polymerase activity through the RRM1 and RRM2 functional domains.

#### hnRNPH1 Interacts with Viral RNA of NP, PB1, and PA but Not PB2

**Figure 4:**
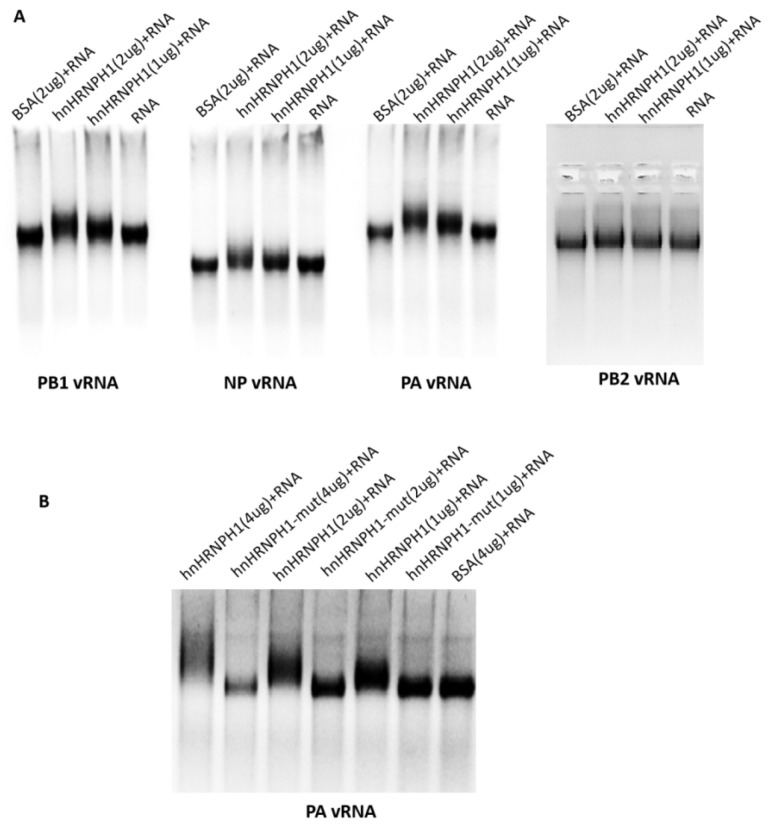
Evaluation of the binding ability of the hnRNPH1 with viral RNA of NP, PB1, PA, and PB2 by MESA. Interaction between hnRNPH1 and vRNA of NP, PB1, PA, and PB2 by EMSA (**A**); Interaction between RRM1 and RRM2 mutated hnRNPH1 and vRNA of PA by EMSA (**B**). vRNA of PB2, PB1, PA, and NP genes was transcribed in vitro. hnRNPH1 and the mutant proteins were expressed and purified in BL21 cells. 1 μg vRNA samples were incubated with 0–4 μg purified hnRNPH1 or BSA proteins as control for 60 min at 23 °C in EMSA/Gel-Shift buffer; the RNA mobility shift was tested through electrophoresis.

To further explore whether hnRNPH1 directly binds influenza virus vRNA to inhibit viral replication, we examined the interaction between hnRNPH1 and influenza viral RNA of vRNPs (PB2, PB1, PA, and NP) using electrophoretic mobility shift assay EMSA. The recombinant hnRNPH1 was expressed and purified, as described previously. The vRNAs of PB1, PB2, PA, and NP were transcribed from the cDNA of the H1N1 influenza virus in vitro. The purified hnRNPH1 protein was incubated with the vRNAs of PB1, PB2, PA, and NP, respectively. The results of EMSA are shown in Figure 4A. In contrast to the BSA+RNA and RNA controls, the vRNA of NP, PB1, and PA lagged clearly after adding the hnRNPH1 protein, except for PB2. Meanwhile, the lagging phenomenon has a dose-dependent manner with the hnRNPH1 protein, which suggests that hnRNPH1 binds to the influenza virus vRNA of the NP, PB1, and PA genes but not PB2.

To further determine the role of RRM of hnRNPH1 on influenza viral RNA-binding capacity, the recombinant mutant protein hnRNPH1 (RRM1+RRM2-WYFY/AAAA) was expressed and purified, then incubated with PA vRNA, and then subjected to EMSA analysis. The results showed that the mutant protein-binding capacity was clearly lower than the parental hnRNPH1 protein (Figure 4B), which confirmed that hnRNPH1 binds to influenza virus vRNA through the RNA-binding function of RRM1 and RRM2.

### 3.4. The Members of hnRNP H Family Inhibit Polymerase Activity of Influenza Virus

**Figure 5:**
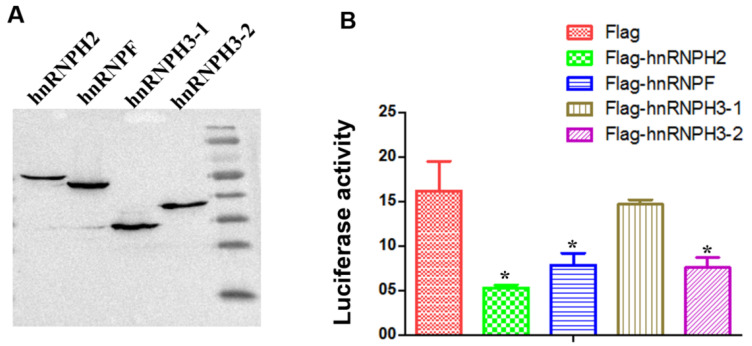
The members of the hnRNP H family inhibit the polymerase activity of the influenza virus. The expression of the hnRNP H family protein in transfected 293T cells (**A**); The relative polymerase activity of H1N1 with overexpressed hnRNP H family proteins in a minigenome assay (**B**). * indicates significant difference between control and treated group (*p* < 0.05).

The HNRNPF/H family of heterogeneous nuclear RNA proteins contains hnRNPH1, hnRNPF, hnRNPH2, and hnRNPH3 members, which are involved in mRNA processing and exhibit extensive sequence homology. To explore whether all the HNRNPF/H family proteins inhibit the polymerase activity of the influenza virus, the human hnRNP F, hnRNP H2, hnRNP H3-1, hnRNP H3-2 were cloned and tested in a minigenome assay. The results showed that hnRNP F, hnRNP H2, and hnRNP H3-2 significantly inhibited the polymerase activity of the influenza virus, suggesting that the hnRNP H family, except for the hnRNP H3-1, are a negative regulator for influenza virus replication (Figure 5).

## 4. Discussion

hnRNPH1 protein is one of the abundant proteins in the nucleus of eukaryotic cells. It participates in the cutting, processing, and transportation of host cell mRNA precursors [21]. The protein mainly binds the intron region to form a stable RNA structure, allowing RNA splices to cut introns [20]. The influenza virus is an RNA virus that replicates in the nucleus of the host cell. During viral replication, there is a large number of viral RNA and proteins in the nucleus. For the host cells, there are relevant proteins or mechanisms in the nucleus that recognize or inhibit the replication of the influenza virus to maintain the homeostasis of the cells [22,23].

A recent study showed that hnRNPH1 binds the genomic RNA of yellow fever virus (YFV), and hnRNPH1 is relocated to the cytoplasm from the nucleus to support virus replication. However, in this study, we found that the overexpressed hnRNPH1 protein inhibited the replication of the H1N1 influenza virus, and after interfering with the hnRNPH1 protein in the cell, the replication of the influenza virus was significantly higher than that in the control group. These results indicated that the hnRNPH1 protein inhibits the replication of the influenza virus.

After the influenza virus enters the cell, its viral RNPs will be transported from the cytoplasm to the nucleus for gene transcription and replication [24]. To explore whether the hnRNPH1 protein inhibits the polymerase activity of the influenza virus, a minigenome polymerase luciferase assay was conducted; it was found that the overexpressed hnRNPH1 protein significantly inhibited the activity of influenza virus polymerase. In order to further verify the functional region of the hnRNPH1 protein on the inhibition of influenza virus, the C-terminus and N-terminus of the protein were truncated, respectively. It was found that the main functional region set of the hnRNPH1 protein was at the N-terminus. The N-terminus of the hnRNPH1 protein contains RRM1 and RRM2 regions, in which four key amino acids, tryptophan and tyrosine in the RRM1 region and phenylalanine and tyrosine in RRM2, determine the RNA-binding ability of hnRPH1 [25]. Mutation of the key amino acid sites of RRM1 and RRM2 abolished the inhibition of polymerase activity of the influenza virus, which suggested that the RNA-binding ability of hnRPH1 is critical for its inhibition of replication of the influenza virus.

Given that hnRNPH1 is an RNA-binding protein and to explore whether hnRNPH1 directly binds to viral RNA, an EMSA assay was conducted. The results showed that the hnRNPH1 protein could bind NP, PB1, and PA vRNA, but not the PB2 vRNA of the influenza virus. Moreover, mutation of the key amino acid sites of RRM1 and RRM2 abolished the PA vRNA-binding ability of hnRNPH1 in EMSA assay, which suggested that hnRNPH1 inhibits the replication of the influenza virus by directly binding the vRNA of the influenza virus. The hnRNP H family proteins bind nucleic acid region containing G-quadruplex [26,27]. The vRNA of WSN/H1 PB1 and PA genes contain G-quadruplex region, the PB2 and NP vRNAs do not contain G-quadruplex. It is rational that hnRNPH1 did not bind PB2 vRNA in the EMSA, while the reason that hnRNPH1 binds NP vRNA without the G-quadruplex motif remains unclear. It is possible that hnRNPH1 could bind certain nucleic acid motifs besides G-quadruplex.

hnRNPH1 plays important roles in host pre-mRNA alternative splicing regulation and mRNA stabilization. In this study, we found that hnRNPH1 only binds the vRNA of the influenza virus in the nucleus at the early stage of infection, after 24 h post-infection. The vRNPs were exported from the nucleus, but hnRNPH1 was still retained in the nucleus, suggesting that hnRNP inhibits influenza virus replication at an early stage. In addition, the role of hnRNPH1 in influenza virus replication and host immune responses needs to be further verified in an animal model.

## 5. Conclusions

In conclusion, the hnRNPH1 protein inhibits the polymerase activity of the influenza virus by binding the vRNA of the influenza virus through the RRM1 and RRM2 regions. The hnRNPH family proteins have similar functions in inhibiting the polymerase activity of the influenza virus.

## Data Availability

The original contributions presented in this study are included in the article. Further inquiries can be directed to the corresponding author.
